# Annotating and detecting phenotypic information for chronic obstructive pulmonary disease

**DOI:** 10.1093/jamiaopen/ooz009

**Published:** 2019-04-26

**Authors:** Meizhi Ju, Andrea D Short, Paul Thompson, Nawar Diar Bakerly, Georgios V Gkoutos, Loukia Tsaprouni, Sophia Ananiadou

**Affiliations:** 1 National Centre for Text Mining, School of Computer Science, The University of Manchester, Manchester, UK; 2 Faculty of Biology, Medicine and Health, The University of Manchester, Manchester, UK; 3 Salford Royal NHS Foundation Trust; and School of Health Sciences, The University of Manchester, Manchester, UK; 4 College of Medical and Dental Sciences, Institute of Cancer and Genomic Sciences, Centre for Computational Biology, University of Birmingham, Birmingham, UK; 5 Institute of Translational Medicine, University Hospitals Birmingham NHS Foundation Trust, Birmingham, UK; 6 MRC Health Data Research UK (HDR UK); 7 NIHR Experimental Cancer Medicine Centre, Birmingham, UK; 8 NIHR Surgical Reconstruction and Microbiology Research Centre, Birmingham, UK; 9 NIHR Biomedical Research Centre, Birmingham, UK; 10 School of Health Sciences, Centre for Life and Sport Sciences, Birmingham City University, Birmingham, UK

**Keywords:** chronic obstructive pulmonary disease, text mining, natural language processing, phenotype, information extraction

## Abstract

**Objectives:**

Chronic obstructive pulmonary disease (COPD) phenotypes cover a range of lung abnormalities. To allow text mining methods to identify pertinent and potentially complex information about these phenotypes from textual data, we have developed a novel annotated corpus, which we use to train a neural network-based named entity recognizer to detect fine-grained COPD phenotypic information.

**Materials and methods:**

Since COPD phenotype descriptions often mention other concepts within them (proteins, treatments, etc.), our corpus annotations include both outermost phenotype descriptions and concepts nested within them. Our neural layered bidirectional long short-term memory conditional random field (BiLSTM-CRF) network firstly recognizes nested mentions, which are fed into subsequent BiLSTM-CRF layers, to help to recognize enclosing phenotype mentions.

**Results:**

Our corpus of 30 full papers (available at: http://www.nactem.ac.uk/COPD) is annotated by experts with 27 030 phenotype-related concept mentions, most of which are automatically linked to UMLS Metathesaurus concepts. When trained using the corpus, our BiLSTM-CRF network outperforms other popular approaches in recognizing detailed phenotypic information.

**Discussion:**

Information extracted by our method can facilitate efficient location and exploration of detailed information about phenotypes, for example, those specifically concerning reactions to treatments.

**Conclusion:**

The importance of our corpus for developing methods to extract fine-grained information about COPD phenotypes is demonstrated through its successful use to train a layered BiLSTM-CRF network to extract phenotypic information at various levels of granularity. The minimal human intervention needed for training should permit ready adaption to extracting phenotypic information about other diseases.

## INTRODUCTION

Chronic obstructive pulmonary disease (COPD) is “a common, preventable, and treatable disease that is characterized by persistent respiratory symptoms and airflow limitation that is due to airway and/or alveolar abnormalities usually caused by significant exposure to noxious particular gases.”^1^ It is rapidly becoming one of the major causes of morbidity and mortality worldwide.^2^ COPD is a multifactorial and heterogeneous disease and not every patient responds to all available drugs.^3–5^ Due to the high prevalence and heterogeneity of COPD, improved deep phenotyping strategies are required. Such in-depth phenotyping can pave the way for personalized treatment regimens,^6^ ensuring that the most suitable therapies are provided.^7^^,^^8^ A phenotype can be broadly defined as “any observable characteristic of an organism,”^9^ while a COPD phenotype can be more specifically defined as “a single or combination of disease attributes that describe differences between individuals with COPD as they relate to meaningful outcomes (symptoms, exacerbations, response to therapy, rate of disease progression, or death).”^10^ Identifying such phenotypes (also described as phenotypic traits) allows grouping of patients according to their prognostic and therapeutic characteristics.^10^ Early classification of the COPD subtype will facilitate superior healthcare provision and early intervention where it is most required—for example, patients with rapid disease progression or frequent exacerbations.

Various textual sources constitute vital sources of COPD evidence, by providing information about phenotypes, characteristics, and treatment regimens. Although pinpointing relevant information in large, heterogeneous text repositories can be time-consuming, applying text mining (TM) techniques to semantically analyze these repositories^11^ can significantly reduce the time needed by clinicians and researchers for tasks such as finding relationships amongst concepts (eg, genotype-phenotype,^12^^,^^13^ gene-disease,^14–16^ and disease-phenotype^17^^,^^18^), diagnosis categorization^19^ or recruiting patients for trials and studies.^20^^,^^21^ To enhance automatic semantic analysis of COPD-related text, the contributions of this article are two-fold:

1. We have created a novel corpus of 30 full-text articles, annotated by experts with named entities relating to COPD phenotypes. The fine-grained annotation scheme aims to account for the potentially complex, nested nature of phenotype descriptions. We automatically enrich the annotations with links to UMLS Metathesaurus concepts. The corpus is freely available (http://www.nactem.ac.uk/COPD) to stimulate development of named entity recognition (NER) tools for COPD phenotypic information.
2. We demonstrate the utility of the corpus by using the expert-added annotations to train a high-performance neural network-based entity recognizer, which exploits nested annotations to accurately detect detailed information relating to COPD phenotypes.

**Figure 1.**
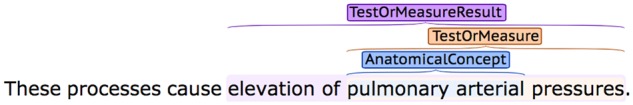
Example of a phenotype that includes other concepts nested within it.

The potential complexity of COPD phenotype descriptions, and how our annotation scheme handles them, is exemplified in Figure 1, where the phrase *elevation of pulmonary arterial pressures* is identified as a phenotype, and is assigned the category *TestOrMeasureResult,* since it describes the outcome of a measurement. Analyzing the internal structure of this phenotype reveals the specific measurement undertaken (*pulmonary arterial pressures*) and anatomical entity involved (*pulmonary artery*). Our annotations correspond to both complete phrases that constitute COPD phenotypes and other types of concepts frequently mentioned within them, and/or within their context. Such embedding (nesting) of shorter entity mentions within longer (outermost) phenotype descriptions is fairly common (29% of our corpus annotations are embedded).

The detailed nature of our annotations aims to facilitate the development of automated tools supporting the exploration of COPD phenotypic information in text from multiple perspectives. This will allow not only the location and categorization of COPD phenotypes, including those identified through tests, or those constituting risk-raising individual behaviors (eg, smoking) but will also permit detailed investigations about the nature of these phenotypes, including finding those affecting specific anatomical locations, or those concerning different results of specific tests. Furthermore, our enrichment of the annotations by applying an automatic normalization method helps to link different ways of mentioning the same concept. This can facilitate search at the concept level, such that searching for the condition *dyspnea* would also retrieve documents mentioning *shortness of breath.*

To demonstrate the full potential of the corpus for developing NER tools, our neural network-based method is specifically designed to recognize nested and outermost entities. In particular, information about nested mentions is used to improve the accuracy of outermost phenotype recognition, without external knowledge resources. To our knowledge, this is the first attempt to apply such an approach to detecting phenotypic information.

## RELATED WORK

### Annotated corpora

Several existing annotated corpora contain entity annotations relevant to phenotype recognition, including biomedical abstracts or articles,^22–24^ medical case reports,^25^ and clinical records.^26^^,^^27^ Certain corpora are also annotated with relations between disorders and other types of concepts.^28^^,^^29^ For example, the phenotype phrase *upper lobe emphysema* may be split into Condition (*emphysema*) and Locus (*upper lobe*), linked by a *has_location* relation.^28^ Such fine-grained analyses allow the potentially complex structure of phenotypes to be exploited to perform more targeted queries, for example, to locate all phenotypes affecting a particular body part. While in most cases, annotations corresponding to phenotypes have rather coarse-grained labels, like *Disease, Disorder,* or *Problem*, a more fine-grained annotation scheme for phenotypes of congestive heart failure (CHF)^30^ distinguishes *Causes, Risk Factors, Non-traditional risk factors,* and *Signs and symptoms.*

The annotations in several corpora^23^^,^^26^^,^^31^^,^^32^ link each annotated entity to a unique concept identifier in a domain-specific terminological resource. Some such resources cover a wide range of medical and biomedical concepts,^33^^,^^34^ while others are specialized for diseases and/or phenotypes.^35–37^ These links can facilitate the development of normalization methods,^31^^,^^38–41^ which automatically assign a concept identifier in a given terminological resource to each entity, to link together variant concept mentions.

### Named entity recognition for COPD

Previous approaches to phenotype NER have included dictionary-based lookup,^42–45^ possibly coupled with rules to improve accuracy and/or to handle the potentially complex structure of phenotype descriptions.^46–50^ Whilst some such approaches perform poorly on phenotype recognition,^51^ an optimized combination of the outputs of these methods can be beneficial.^52^ However, combining or replacing rules with machine learning (ML) tends to achieve superior performance.^53–55^

Conventional ML approaches such as conditional random fields (CRFs) have been applied to many NER tasks, including detecting CHF phenotypes^30^ and recognizing nested entities.^56–58^ CRF-based models generally require humans to perform feature engineering for each new task, to determine the optimal set of textual features for predicting entities. Features include semantic information from domain-specific terminological resources or the output of linguistic processing tools, which can be time-consuming to apply to huge document collections.

Recently, however, representational methods have improved phenotype extraction performance^59–61^ by using word embeddings*,* which remove the need for hand-crafted feature engineering, linguistic processing or terminological resources,^62^^,^^63^ and character embeddings, which encode word morphology information.

Combined with embeddings, advanced deep learning methods can produce high-performance NER systems.^64–66^ Recurrent neural networks (RNNs)^67^ are effective for various natural language processing tasks,^68^ while specializations such as long short-term memory networks (LSTMs)^69^ and gated recurrent units (GRUs)^70^ are particularly effective, since they introduce gating mechanisms to handle textual contexts with long dependencies, which can be highly important for NER.^71^ Bidirectional versions (eg, bidirectional long short-term memory [BiLSTMs]) use information from both left and right contexts, to further boost performance.^72^^,^^73^

In addition to “standard” NER tasks, neural network methods have been applied to nested entity recognition.^74^^,^^75^ Multilayered approaches^76^^,^^77^ use information about entities at a given level of nesting to improve recognition of entities at other levels of nesting. One of these^77^ uses no linguistic features, and outperforms other methods in detecting nested entities for general language and molecular biology.

## METHODS

**Figure 2.**
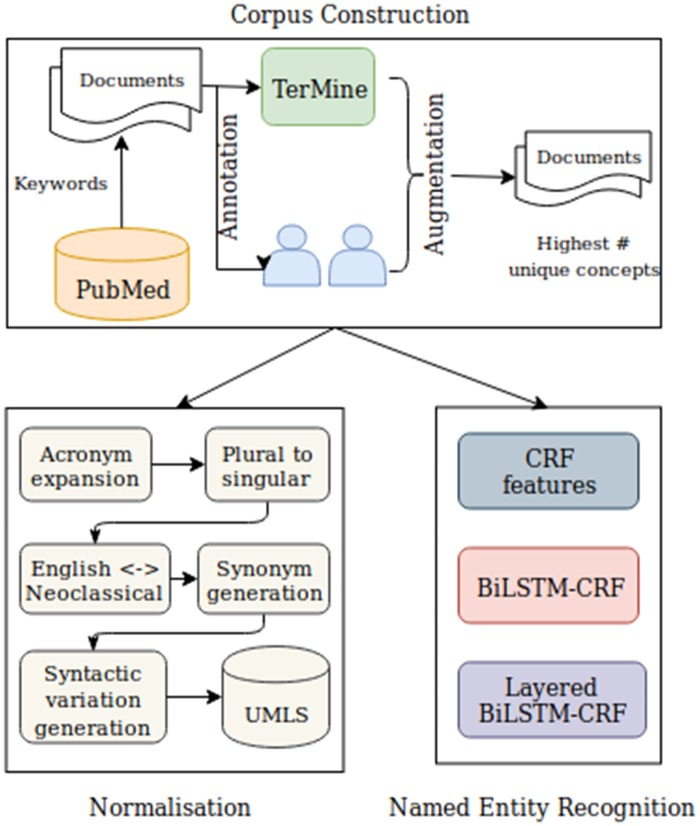
Workflow for annotation and detection of information relating to COPD phenotypes. COPD: chronic obstructive pulmonary disease; CRF: conditional random field.

In this section, we explain the methods used in the various steps of our work (see Figure 2 for an overview). Firstly, we describe the construction and annotation of the corpus. We subsequently explain how the expert-added annotations were enriched using an automatic normalization method to link them to UMLS concepts. Finally, we describe the NER methods that were applied to create a named entity recognizer for COPD.

### Corpus construction

Information about COPD phenotypes may occur in various documents, including clinical records and academic articles. However, the availability of clinical record corpora is restricted, and they tend to be US-centric.^78^^,^^79^ To avoid bias toward practices of a particular country, we decided to create a corpus of scientific articles from various COPD-relevant journals. As previous work has shown that TM tools trained on one text type can be applied to texts with different characteristics,^30^^,^^80^ it is intended that tools trained on our corpus may be adapted for phenotype extraction from clinical records.

We firstly selected COPD-relevant journals in the PubMed Central Open Access Subset, whose titles contain the following keywords: (*chronic, obstructive, pulmonary, disease, respiratory*, and *lung*); this resulted in the 10 journals shown in Supplementary Appendix S1. We then retrieved all articles within these journals mentioning either *chronic obstructive pulmonary disease* or *COPD* (974 articles). According to limited resources and time, only a subset of these documents could be annotated by our domain experts. We thus attempted to select documents containing the richest and widest COPD phenotype evidence. We firstly applied the automatic term recognition system TerMine^81^ to the set of the COPD guidelines published jointly by the American Thoracic Society and the European Respiratory Society.^82^ The automatically extracted terms were augmented with expert-provided terms to create 1925 different terms representing COPD phenotypes. We then selected the 30 full-text articles with the highest numbers of unique COPD phenotype terms. The number of unique terms in each selected document is shown in Supplementary Appendix S2.

### Annotation scheme

Our annotation scheme^83^ (guidelines available at: http://www.nactem.ac.uk/COPD/download.php) aims to balance simplicity of application with the ability to capture fine details about phenotypes. Only simple text spans, rather than relationships, are annotated, since the latter task can considerably increase annotation burden. However, by using a detailed hierarchy of semantic labels, and allowing entities to be nested within each other, we can capture potential relationships between entities. For example, if a treatment is mentioned within a phenotype statement (*Steroid-induced skeletal muscle atrophy*), then it is likely that the phenotypic manifestation is a side effect of the nested treatment.

**Figure 3.**
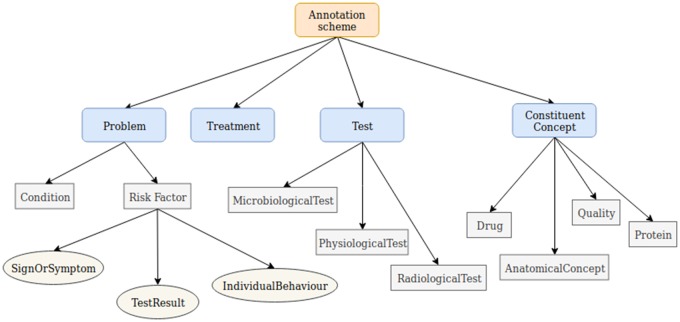
Hierarchical entity annotation scheme for COPD phenotypic information. COPD: chronic obstructive pulmonary disease.

Our scheme (see Table 1 and Figure 3) is inspired by 2 existing schemes. The categories defined in 1 scheme,^29^ that is, *Problem*, *Treatment*, or *Test*, form the core of the scheme, to identify information about COPD phenotypes, their treatment and discovery. Inspired by the fine-grained labels used for CHF phenotypes,^30^ we introduce a hierarchy of more detailed labels under these top-level categories; the most specific labels possible are assigned by annotators. Since phenotype descriptions are typically formed from a combination of different types of concepts, our scheme includes the most common of these, for example, anatomical concepts (*chronic airways obstruction*), proteins (*alpha1 antitrypsin deficiency*), qualities (eg, *decreased COPD exacerbations*), and test results (eg, *reduced FEV1*). These are mainly organized under an additional top-level category, *ConstituentConcept*.

**Table 1.** Descriptions, examples, and counts of each category in the COPD annotation scheme

Type	Description	Examples	Number of concepts
Problem	An overall category for any COPD indicates of concern	COPD exacerbations; past pulmonary TB	2556
Condition	Any disease or medical condition includes COPD comorbidities	emphysema; pulmonary vascular disease; asthma	5119
RiskFactor	A phrase signifying a patient’s increased chances of having COPD	increased levels of the C-reactive protein; alpha1 antitrypsin deficiency	1211
SignOrSymptom	An observable irregularity manifested by a COPD patient	chronic cough; shortness of breath	2065
IndividualBehaviour	A patient’s habits leading to susceptibility of having COPD	smoking for 25 years; exercise-limited patients	194
TestResult	Findings based on COPD-relevant examinations	decrease in rate of lung function; FEV1 45% predicted	685
Treatment	Any medication, therapy, or treatment program	inhaled corticosteroids; oxygen therapy; pulmonary rehabilitation	4337
Test	An overall category for any COPD-relevant examinations or measures/parameters	spirometry, respiratory frequency, FEV1	3576
RadiologicalTest	Any of the radiological tests for detecting COPD	computed tomography scanning; high resolution computed tomography	29
MicrobiologicalTest	An examination of a COPD-relevant specimen	complete blood count; bacterial isolates	11
PhysiologicalTest	A measurement of a COPD patient’s capacity to exercise	6-min walking distance; incremental cardiopulmonary exercise testing	17
ConstituentConcept	An umbrella type for elementary concepts that may form part of a phenotype description; should only be chosen if none of the subtypes below apply	bronchodilation; enhancement of skeletal muscle contractility	5
AnatomicalConcept	A mention pertaining to anatomical entities	lung; heart; pulmonary; hepatic; respiratory airway	2616
Drug	Any drug name; will mostly overlap with treatment	corticosteroids; short-acting bronchodilators	2593
Protein	Any protein name	alpha1 antitrypsin; pro-inflammatory cytokines	820
Quality	Expressions which modify or qualify any of the concepts above	chronic; obstructed; damaged; decreased rate; enhanced; decreased amount	1153

*Abbreviations:* COPD: chronic obstructive pulmonary disease; FEV1: Forced Expiratory Volume.

To increase annotation ease and efficiency, we used Argo,^84^ an interoperable TM platform, to apply a pipeline of pre-existing NER tools to preannotate the corpus with several entity types typically mentioned within phenotypes. The annotators’ task was then limited to reviewing and editing automatically added annotations, or adding longer, spanning annotations corresponding to more complex phenotypes.

To ensure annotation quality and consistency, 6 full-text papers were firstly annotated independently by 2 annotators with medical expertise, and inter-annotator agreement (IAA) rates were calculated. The widely used Cohen's kappa is not suitable here, because it requires the total number of annotated items to be known in advance. Hence, we followed a number of other related efforts^85–87^ by calculating IAA in terms of F-score. The micro-averaged IAA rate was 80.49% F-score, using strict conditions (ie, requiring both annotators’ annotations to match exactly in terms of text span chosen and semantic category). The main areas of disagreement concerned some fine-grained categories within the *Problem* branch of the scheme. In consultation with the annotators, the definitions of these categories were reviewed, and disagreements were discussed and resolved. Taking into account the decisions made, one of the annotators annotated the remaining 24 papers.

### Entity normalization

We automatically normalized annotated entities to unique concept identifiers in the UMLS Metathesaurus,^33^ which covers all entity types in our scheme. We chose the HYPHEN method^41^ because of its flexibility, that is, it can normalize different entity types in documents with varying characteristics to different target terminological resources.^33^^,^^88^

HYPHEN uses a pipeline of different techniques to generate semantically consistent variations of the original entity mention and tries to match these generated variants against existing variants listed in the target terminological resource. The 6 techniques are:

1. Acronym/abbreviation expansion (eg, *Type 2 DM* → *Type 2 diabetes mellitus*).
2. Plural to singular conversion (eg, *alveolar septa* → *alveolar septum*).
3. Generation of English equivalents of Neoclassical compounds (eg, *elevated blood leukocyte counts* → *elevated white blood cell count*).
4. Generation of Neoclassical equivalents of English terms (eg*, pleural inflammation* → *pleuritis*).
5. Syntactic variation generation (eg, *supplemental oxygen* → *oxygen supplementation*).
6. Synonym generation (eg, *worsening pulmonary function* → *deterioration of lung function*).

Table 2 reports on the number and percentage of entities belonging to each category in our corpus that are automatically normalized. For each category, normalization dictionaries were created by filtering the concepts belonging to different UMLS semantic types; these are detailed in Supplementary Appendix S3.

**Table 2.** Number of entities normalized by HYPHEN

Category	Total entities	Number of entities normalized	Percentage of entities normalized
Problem	2556	2151	83.15
Condition	5119	4969	97.07
RiskFactor	1211	942	77.79
SignOrSymptom	2065	1140	55.21
IndividualBehaviour	194	124	63.92
TestOrMesureResult	685	259	37.81
Treatment	4337	3775	87.04
TestOrMeasure	3576	2609	72.96
AnatomicalConcept	2616	2372	90.67
Drug	2593	2368	91.32
Protein	820	727	87.66
Quality	1153	1015	88.03
Total	26 925	22 451	83.38

As shown in Table 2, HYPHEN normalized a high percentage (83.38%) of entity annotations in the corpus to UMLS concept identifiers. Some examples of successful normalizations are shown in Table 3.

**Table 3.** Sample normalization results

Entity annotation	Semantic category	Mapped UMLS concept
increased PVR	Problem	Increased pulmonary vascular resistance (C1867423)
lung failure	Condition	Pulmonary failure (C0948755)
left atrial	AnatomicalConcept	Left atrium (C0225860)
arm training	Treatment	Upper limb training (C0556501)
spirometric test	TestOrMeasure	Spirometry test (C0037981)
genetic predisposition	RiskFactor	Genetic susceptibility to disease (C1455997)

HYPHEN works well in normalizing entities describing single, straightforward concepts. Although most entity annotations possess such characteristics, performance is lower for categories whose annotations exhibit divergent characteristics. These include *SignOrSymptom,* whose annotations include long, detailed phrases, for example, *daily productive cough for a minimum of 3 months for a minimum of 2 consecutive years* or those mentioning multiple concepts, for example, *coughing and/or corticosteroid-induced osteoporosis.* The most problematic category, *TestOrMeasureResult,* includes mentions with no corresponding UMLS concepts (eg, *negative pleural pressure*), or those including numeric values (eg, *oxygen saturation level 90%*), which cannot be mapped to *high oxygen saturation* (C0852710) without additional processing.

### Named Entity Recognition methods

We used the COPD corpus to train a named entity recognizer which can handle multiple levels of entity nesting.^77^ We adopted an existing neural network architecture^64^ for recognizing “flat” (ie, nonnested) named entities, to form the “building blocks” of our layered model for nested entity recognition. In this architecture, rich representations of word properties were obtained by combining word embeddings^89^ and character-level embeddings. A combination of BiLSTM and CRF was used to detect and classify entities.

Our approach builds upon this architecture, using a stack of multiple BiLSTM-CRF layers, each intended to detect a subset of entities. The input to each layer depends on the output of the previous layer. The input to the first layer consists of word and character-level embeddings for each individual word. The information about all words in each entity detected by this layer is merged into a single unit, whose representation combines information about each individual word in the entity. The merged information is passed to the next layer to aid in recognizing entities with higher levels of nesting. This key feature of our approach aims to account for potential dependencies between entities with different levels of nesting, that is, information about entities with lower nesting levels may provide clues about the presence of higher-level entities that include the nested entities within them.

**Figure 4.**
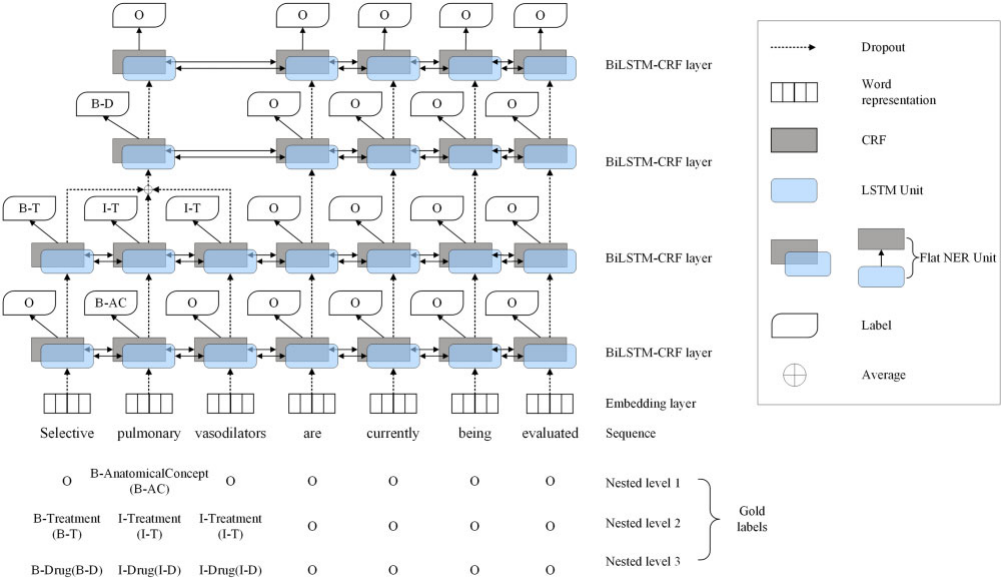
Overview of the layered-BiLSTM-CRF model architecture. B-AC: B-AnatomicalConcept; B-T: B-treatment; I-T: I-treatment; B-D: B-drug; I-D: I-drug.

The method is dynamic—it stacks as many new layers as are necessary to allow all nested entities to be recognized; the method terminates when no entities are discovered by a newly stacked layer. Figure 4 illustrates the model architecture, where annotations are transformed into BIO tagging scheme labels to allow the model to be trained. These labels identify whether each word comes at the (B)eginning, I(nside), or (O)uside of an entity annotation. Although more complex tagging schemes may be used, for example, BIOES, which distinguishes words that constitute S(ingle) word entities, or which come at the E(nd) of multi-word entities, we chose to use BIO to avoid data sparsity problems, since some of our categories include relatively few annotations.

### Baseline models

We firstly randomly split the corpus into 3 different parts—four-fifths for training, one-tenth for development (to tune parameters used by the models using Bayesian optimization^90^), and one-tenth for testing.

Based on previous studies,^73^^,^^91^ deciding on an optimal deep learning model, and whether to combine it with CRF, appears to be influenced by the task at hand. Using the layered architecture outlined above, we trained and evaluated different deep learning models using different algorithms (BiRNN, BiGRU, and BiLSTM), both in isolation and in combination with CRF; we found that the BiLSTM-CRF model attains the best results (see Supplementary Appendix S4 for performance statistics and tuned hyperparameter values).

We also compared our layered BiLSTM-CRF model to a CRF model and a “flat” (non-layered) BiLSTM-CRF model; the results of these experiments are shown in Table 4. We used NERSuite^92^ to implement the CRF model, whose features include contextual information, such as n-grams (ie, up to 3 words either side of the entity), parts-of-speech, syntactic chunks, and word base forms.^92^ In contrast, the non-layered BiLSTM-CRF uses only word and character-level embeddings instead of features, as described above.

**Table 4.** Performance of different NER models at different levels of entity nesting

Level	Model	P (%)	R (%)	F (%)
Innermost	CRF	**77.19**	68.78	72.74^a^
	BiLSTM-CRF	73.93	**73.38**	**73.56** ^a^
	Layered BiLSTM-CRF	69.79	70.41	70.10
Outermost	CRF	73.63	66.41	69.83^a^
	BiLSTM-CRF	**75.61**	67.35	71.24^a^
	Layered BiLSTM-CRF	74.00	**74.54**	**74.27**
All	CRF	75.44	67.61	71.31^a^
	BiLSTM-CRF	74.71	70.42	72.50^a^
	Layered BiLSTM-CRF	**77.02**	**75.45**	**76.23**

*Note*: For each different level, the best precision (P), recall (R), and F-score (F) amongst the 3 models is shown in bold.

*Abbreviations:* NER: named entity recognition; CRF: conditional random field.

^a^ A significant difference between CRF and (flat) BiLSTM-CRF models at *P* < .05. Since the layered BiLSTM-CRF takes as input different entities than the baseline models (ie, all entities vs innermost or outermost entities), we did not apply significance testing between layered and flat models.

### Experimental settings

We conducted experiments in a single run rather than using cross-validation, in order to minimize overfitting to the training corpus. Our experiments evaluate performance variations of each model when entities with different levels of nesting are considered. We consider *innermost* entities, *outermost* entities, and *all* entities in the test dataset. Innermost entities are the most deeply nested entities, while outermost entities are non-nested entities. In Figure 1, *elevation of pulmonary arterial pressures* is the outermost entity, while *pulmonary arterial* is the innermost entity. Entities without nesting (eg, *dyspnea*) are included in both the innermost and outermost sets.

For the CRF and non-layered BiLSTM-CRF, we train separate models to recognize only innermost and outermost entities. In contrast, our layered BiLSTM-CRF is trained to recognize entities at all levels of nesting; we evaluate its performance in recognizing different levels of entities by considering outputs of different model layers.

## RESULTS

Table 4 shows the performance of each model. The non-layered BiLSTM-CRF performs best for innermost entities, demonstrating how embeddings can successfully replace the multiple linguistic features used by the CRF. At this level, however, the layered BiLSTM-CRF has lower performance than the non-layered BiLSTM-CRF. For the layered model, we consider only the output of its first layer, which is expected to recognize only innermost entities. However, error analysis revealed that there is actually not a one-to-one correspondence between model layers and entity nesting levels, that is, the first layer sometimes detects entities belonging to other (ie, not innermost) entity levels. Conversely, higher layers of the model may detect entities that belong to the innermost nesting level.

For outermost entities, the non-layered BiLSTM-CRF still outperforms the CRF, reinforcing the advantages of deep learning. However, in contrast to innermost entities, the layered BiLSTM-CRF outperforms the non-layered model in detecting outermost entities. This clearly demonstrates how the layered model’s use of information about lower-level entities improves recognition of higher-level entities.

The higher performance of the layered BiLSTM-CRF for outermost entities also provides evidence that innermost entities are successfully recognized by lower levels of the model. This is confirmed by its superior performance to the other models in detecting all entities in the test dataset. Although there is no exact correspondence between the recognition of specific levels of entities and layers of the model, the complete model is still able to exploit the output of previous layers to achieve a high level of performance in detecting both outermost and nested entities. Detailed performance statistics for the layered BiLSTM-CRF by entity type are provided in Supplementary Appendix S5.

## DISCUSSION

The results achieved by our layered BiLSTM-CRF in recognizing COPD-related information are superior to those achieved by applying the same model to nested entity recognition in well-used corpora from other domains.^77^ This provides evidence that our corpus is suitable for training high-performance ML-based tools, and that automatic recognition of COPD phenotypic information is a feasible task. Moreover, we have shown that detecting COPD phenotype information using deep learning models, which require minimal human intervention for training, can achieve superior performance to more traditional methods requiring time-consuming feature engineering, linguistic processing, and terminological resources. We have furthermore demonstrated that our layered model can achieve superior performance to a “flat” model, by exploiting information about nested entities when detecting the longer entities in which they are embedded.

These outcomes have important implications, in terms of improving the ease of locating phenotypic information in text. In particular, our nested entity detection method not only allows efficient location of COPD phenotype descriptions hidden in large text collections, but it also detects the internal structure of these descriptions. This provides scope to explore and categorize COPD phenotypes in a fine-grained manner. Since our method can be rapidly adapted to detect different types of information, it could be readily applied to find phenotypic information relating to other diseases, given suitably annotated corpora.

**Figure 5.**
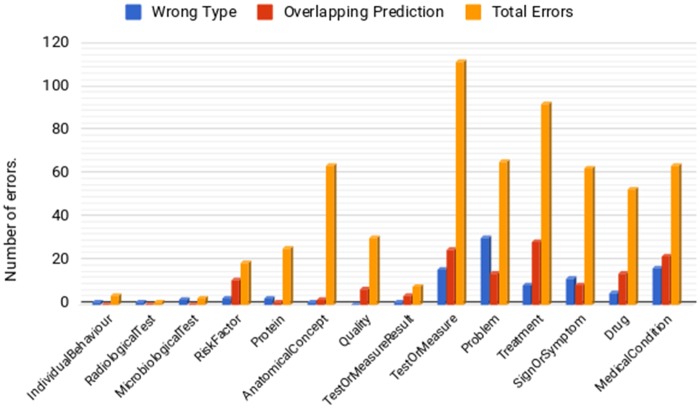
Counts of different types of errors for each semantic type.

Error analysis of our NER results reveals that about 17% of erroneous entities have the correct text span, but the wrong semantic category. Figure 5 provides detailed error statistics for each semantic type, revealing that *Problem* is the most frequently misclassified category; these entities are mainly misclassified as either *MedicalCondition* or *SignOrSymptom*. Conversely, *MedicalCondition* entities are mostly misclassified as *Problem.* Such errors are possibly due to the fine-grained, hierarchical structure of our annotation scheme; the often subtle differences between similar categories may be difficult for the computer to distinguish. A further 23% of errors (most frequently *Treatment* and *TestOrMeasure* entities) concern cases where the model assigns the correct category, but the wrong text span (ie, it partially overlaps with the correct span). This may be due to the heterogeneity of phenotype descriptions, which can include mentions of various concept types, and which may or may not include modifier phrases. However, it is significant that in around 40% of the erroneous cases, the model can successfully detect the presence of entities, and categorize them correctly. Thus, even if the span is not completely correct, the model can find documents mentioning relevant entities, and allow examination of the context surrounding these entities.

## CONCLUSION

We have described the construction of a novel corpus of full-text articles about COPD, annotated using a scheme that identifies pertinent information about COPD phenotypes, in which nested entity annotations make explicit the internal structure of potentially complex phenotype descriptions. The corpus is intended to assist the development of novel NER approaches to COPD phenotype recognition. The annotations were enriched using a high-performance normalization method to link the majority of them to UMLS Metathesaurus concepts.

We demonstrated the utility of the corpus by using it to train a deep learning-based NER model, which is designed to recognize entities with different levels of nesting and, in contrast to many ML-based models, relies on neither linguistic features nor external knowledge resources.

The detailed, fine-grained information about COPD phenotypes output by our model will facilitate development of semantic search systems for textual repositories, to pinpoint phenotype-relevant information, for example, to identify treatment regimens and investigate their relative effectiveness in different disease phenotypes. The ease of applying the NER model to newly available data will facilitate repeated interrogation of relevant data sources, allowing tracking of disease progression in individuals, and alerting clinicians to changes in disease pattern. Resolving entities to UMLS Metathesaurus concepts will facilitate concept-level search, in which all mentions of a concept of interest can be found automatically, regardless of the actual words or phrases used to describe them.

As future work, we will extend our framework to increase the complexity of the information extracted, inspired by recent work^93^^,^^94^ applying deep neural network models to medical relationship extraction. We will also apply our method to clinical records and to the detection of phenotypes of other diseases. This will reinforce the importance of our method in helping to enhance clinical phenotyping and early classification of disease subtype, providing a means of early, accurate diagnosis, and personalized treatment regimens for patients.

## SUPPLEMENTARY MATERIAL

Supplementary material is available at *Journal of the American Medical Informatics Association* online.

## CONTRIBUTORS

M.J. ran set up, ran the named entity recognition experiments, and analyzed their output. A.D.S., P.T., N.D.B., L.T., and G.V.G. contributed toward designing and refining the annotation scheme. A.D.S. and L.T. performed the annotation of the corpus. P.T. ran the normalization method and analyzed the output. P.T., M.J., and S.A. drafted the manuscript. N.D.B., L.T., and G.V.G. revised the manuscript. All authors read and approved the final version of the manuscript. S.A. supervised all steps of the work.

## Supplementary Material

Supplement_Material_ooz009
