# Supplementary material for: Annotating and detecting phenotypic information for chronic obstructive pulmonary disease
Source: JAMIA Open. 2019 Apr 26;2(2):261–71. doi: 10.1093/jamiaopen/ooz009 (PMC6951876; doi:10.1093/jamiaopen/ooz009)
Supplement: Supplement_Material_ooz009 [file supplement_material_ooz009.zip › APPENDIX 1.docx]

**APPENDIX 1 – PMC JOURNALS RELEVANT TO COPD**

In Table 1, the details of all COPD relevant journals included in the corpus collection stage are shown. The journals were selected from the PubMed Central Open Access Subset, by finding journals whose titles contained the following keywords: *chronic, obstructive, pulmonary, disease, respiratory* and *lung*.

For each journal, we show the international standardised serial number (ISSN) for the journal, the journal title and the range of volumes within the journal that was searched for COPD-relevant articles.

**Table 1:** COPD-relevant journals selected as the basis for creating the COPD corpus.

| **ISSN** | **Title** | **Searched Volumes in PMC** | |
| --- | --- | --- | --- |
|  |  | **Latest** | **Earliest** |
| 1471-2466 | BMC Pulmonary Medicine | v.14 2014 | v.1 2001 |
| 1541-7891 | Cardiopulmonary Physical Therapy Journal | v.24(3) Sep 2013 | v.19 2008 |
| 1179-5484 | Clinical Medicine Insights. Circulatory, Respiratory and Pulmonary Medicine (v.2;2008) | v.8 2014 | v.4 2010 |
| 1178-1157 | Clinical Medicine. Circulatory, Respiratory and Pulmonary Medicine - now published as Clinical Medicine Insights. Circulatory, Respiratory and Pulmonary Medicine | v.2 2008 | v.2 2008 |
| 1176-9106 | International Journal of Chronic Obstructive Pulmonary Disease | v.9 2014 | v.1 2006 |
| 2045-8932 | Pulmonary Circulation | v.3(2) Apr-Jun 2013 | v.1 2011 |
| 2090-1836 | Pulmonary Medicine | v.2014 2014 | v.2010 2010 |
| 2282-8419 | Heart, Lung and Vessels | v.5(4) 2013 | v.5 2013 |
| 1545-1151 | Preventing Chronic Disease | v.11 2014 | v.1 2014 |
| 2040-6223 | Therapeutic Advances in Chronic Disease | v.5(1) Mar 2014 | v.1 2010 |
