# Supplementary material for: Annotating and detecting phenotypic information for chronic obstructive pulmonary disease
Source: JAMIA Open. 2019 Apr 26;2(2):261–71. doi: 10.1093/jamiaopen/ooz009 (PMC6951876; doi:10.1093/jamiaopen/ooz009)
Supplement: Supplement_Material_ooz009 [file supplement_material_ooz009.zip › APPENDIX 2.docx]

**APPENDIX 2 – TOP-RANKED COPD-RELEVANT ARTICLES**

COPD-relevant articles were selected from the COPD-relevant journals (shown in Appendix 1) by filtering those articles mentioning either chronic obstructive pulmonary disease or COPD (a total of 974 articles). As the basis for the annotated corpus, we selected the articles containing the richest and widest COPD phenotype evidence. We firstly applied the automatic term recognition system TerMine to the set of the COPD guidelines published jointly by the American Thoracic Society and the European Respiratory Society. The automatically extracted terms were augmented with expert-provided terms to create a total of 1,925 different terms representing COPD phenotypes. We then selected the 30 full-text papers with the highest numbers of unique COPD phenotype terms.

Table 1 shows the 30 top-ranked articles according to the above criteria. For each article, we show the PMCID, the number of unique COPD phenotype terms found within the article, and the total number of COPD phenotype term mentions in the article.

**Table 1:** Top 30 articles in selected COPD journals, ranked according to the number of unique COPD relevant terms mentioned within them.

| **Ranking** | **PMCID** | **Number of unique COPD phenotype terms** | **Total number of COPD phenotype term mentions** |
| --- | --- | --- | --- |
| 1 | PMC2699974 | 168 | 630 |
| 2 | PMC3224650 | 137 | 643 |
| 3 | PMC2707802 | 136 | 692 |
| 4 | PMC3109340 | 135 | 629 |
| 5 | PMC3107696 | 132 | 600 |
| 6 | PMC3986113 | 128 | 727 |
| 7 | PMC2740954 | 126 | 787 |
| 8 | PMC2699821 | 118 | 943 |
| 9 | PMC2650603 | 114 | 633 |
| 10 | PMC2707151 | 112 | 649 |
| 11 | PMC2754087 | 112 | 602 |
| 12 | PMC3641739 | 112 | 631 |
| 13 | PMC2650593 | 111 | 449 |
| 14 | PMC2528217 | 110 | 739 |
| 15 | PMC3933347 | 110 | 737 |
| 16 | PMC2650592 | 108 | 559 |
| 17 | PMC2707800 | 107 | 617 |
| 18 | PMC3869834 | 107 | 420 |
| 19 | PMC3034289 | 106 | 487 |
| 20 | PMC3437672 | 106 | 639 |
| 21 | PMC2898088 | 103 | 579 |
| 22 | PMC2650600 | 102 | 900 |
| 23 | PMC3908831 | 102 | 477 |
| 24 | PMC1764756 | 101 | 316 |
| 25 | PMC2706597 | 99 | 683 |
| 26 | PMC2695202 | 99 | 765 |
| 27 | PMC3212861 | 96 | 645 |
| 28 | PMC3653890 | 95 | 511 |
| 29 | PMC2898089 | 95 | 574 |
| 30 | PMC2528206 | 95 | 1110 |
