# Supplementary material for: Annotating and detecting phenotypic information for chronic obstructive pulmonary disease
Source: JAMIA Open. 2019 Apr 26;2(2):261–71. doi: 10.1093/jamiaopen/ooz009 (PMC6951876; doi:10.1093/jamiaopen/ooz009)
Supplement: Supplement_Material_ooz009 [file supplement_material_ooz009.zip › APPENDIX 3.docx]

### APPENDIX 3 – NORMALISATION DICTIONARIES

Tables 1-8 list the UMLS semantic types used to create the normalisation dictionaries for use by HYPHEN for the various different semantic entity types.

**Table 1:** UMLS semantic types used to create normalisation dictionaries for Problem, Condition and SignOrSymptom.

| **Code** | **Label** | **Code** | **Label** |
| --- | --- | --- | --- |
| T020 | Acquired Abnormality | T037 | Injury or Poisoning |
| T190 | Anatomical Abnormality | T048 | Mental or Behavioral Dysfunction |
| T049 | Cell or Molecular Dysfunction | T191 | Neoplastic Process |
| T019 | Congenital Abnormality | T046 | Pathologic Function |
| T047 | Disease or Syndrome | T184 | Sign or Symptom |
| T050 | Experimental Model of Disease | T033 | Finding |

**Table 2:** UMLS semantic types used to create normalisation dictionaries for AnatomicalConcept.

| **Code** | **Label** | **Code** | **Label** |
| --- | --- | --- | --- |
| T029 | Body Location or Region | T017 | Anatomical Structure |
| T023 | Body Part, Organ, or Organ Component | T025 | Cell |
| T030 | Body Space or Junction | T026 | Cell Component |
| T031 | Body Substance | T018 | Embryonic Structure |
| T022 | Body System | T024 | Tissue |
| T021 | Fully Formed Anatomical Structure |  |  |

**Table 3:** UMLS semantic types used to create normalisation dictionaries for Drug.

| **Code** | **Label** | **Code** | **Label** |
| --- | --- | --- | --- |
| T116 | Amino Acid, Peptide, or Protein | T125 | Hormone |
| T195 | Antibiotic | T129 | Immunologic Factor |
| T123 | Biologically Active Substance | T130 | Indicator, Reagent, or Diagnostic Aid |
| T122 | Biomedical or Dental Material | T197 | Inorganic Chemical |
| T118 | Carbohydrate | T119 | Lipid |
| T103 | Chemical | T124 | Neuroreactive Substance or Biogenic Amine |
| T120 | Chemical Viewed Functionally | T114 | Nucleic Acid, Nucleoside, or Nucleotide |
| T104 | Chemical Viewed Structurally | T109 | Organic Chemical |
| T200 | Clinical Drug | T115 | Organophosphorus Compound |
| T111 | Eicosanoid | T121 | Pharmacologic Substance |
| T196 | Element, Ion, or Isotope | T192 | Receptor |
| T126 | Enzyme | T110 | Steroid |
| T131 | Hazardous or Poisonous Substance | T127 | Vitamin |

**Table 4:** UMLS semantic types used to create normalisation dictionaries for Treatment.

| **Code** | **Label** | **Code** | **Label** |
| --- | --- | --- | --- |
| T116 | Amino Acid, Peptide, or Protein | T114 | Nucleic Acid, Nucleoside, or Nucleotide |
| T195 | Antibiotic | T109 | Organic Chemical |
| T123 | Biologically Active Substance | T115 | Organophosphorus Compound |
| T122 | Biomedical or Dental Material | T121 | Pharmacologic Substance |
| T118 | Carbohydrate | T192 | Receptor |
| T103 | Chemical | T110 | Steroid |
| T120 | Chemical Viewed Functionally | T127 | Vitamin |
| T104 | Chemical Viewed Structurally | T060 | Diagnostic Procedure |
| T200 | Clinical Drug | T065 | Educational Activity |
| T111 | Eicosanoid | T058 | Health Care Activity |
| T196 | Element, Ion, or Isotope | T059 | Laboratory Procedure |
| T126 | Enzyme | T063 | Molecular Biology Research Technique |
| T131 | Hazardous or Poisonous Substance | T062 | Research Activity |
| T125 | Hormone | T061 | Therapeutic or Preventive Procedure |
| T129 | Immunologic Factor | T093 | Health Care Related Organization |
| T130 | Indicator, Reagent, or Diagnostic Aid | T203 | Drug Delivery Device |
| T197 | Inorganic Chemical | T074 | Medical Device |
| T119 | Lipid | T075 | Research Device |
| T124 | Neuroreactive Substance or Biogenic Amine | T168 | Food |

**Table 5:** UMLS semantic types used to create normalisation dictionaries for TestOrMeasure and TestOrMeasureResult.

| **Code** | **Label** | **Code** | **Label** |
| --- | --- | --- | --- |
| T060 | Diagnostic Procedure | T201 | Clinical Attribute |
| T065 | Educational Activity | T032 | Organism Attribute |
| T058 | Health Care Activity | T034 | Laboratory or Test Result |
| T059 | Laboratory Procedure | T078 | Idea or Concept |
| T063 | Molecular Biology Research Technique | T170 | Intellectual Product |
| T062 | Research Activity | T042 | Organ or Tissue Function |
| T031 | Body substance | T080 | Qualitative Concept |
| T033 | Finding | T079 | Temporal Concept |

**Table 6:** UMLS semantic types used to create normalisation dictionaries for RiskFactor and IndividulaBehaviour.

| **Code** | **Label** | **Code** | **Label** |
| --- | --- | --- | --- |
| T052 | Activity | T047 | Disease or Syndrome |
| T053 | Behavior | T050 | Experimental Model of Disease |
| T056 | Daily or Recreational Activity | T033 | Finding |
| T051 | Event | T037 | Injury or Poisoning |
| T055 | Individual Behavior | T048 | Mental or Behavioral Dysfunction |
| T057 | Occupational Activity | T191 | Neoplastic Process |
| T054 | Social Behavior | T046 | Pathologic Function |
| T020 | Acquired | T184 | Sign or Symptom |
| T190 | Anatomical Abnormality | T131 | Hazardous or Poisonous Substance |
| T049 | Cell or Molecular Dysfunction | T069 | Environmental Effect of Humans |
| T019 | Congenital Abnormality | T123 | Biologically Active Substance |

**Table 7:** UMLS semantic types used to create normalisation dictionaries for Protein.

| **Code** | **Label** |
| --- | --- |
| T116 | Amino Acid, Peptide, or Protein |

**Table 8:** UMLS semantic types used to create normalisation dictionaries for Quality.

| **Code** | **Label** | **Code** | **Label** |
| --- | --- | --- | --- |
| T185 | Classification | T077 | Conceptual Entity |
| T169 | Functional Concept | T102 | Group Attribute |
| T078 | Idea or Concept | T170 | Intellectual Product |
| T171 | Language | T080 | Qualitative Concept |
| T081 | Quantitative Concept | T089 | Regulation or Law |
| T082 | Spatial Concept | T079 | Temporal Concept |
| T033 | Finding |  |  |
