# Supplementary material for: Annotating and detecting phenotypic information for chronic obstructive pulmonary disease
Source: JAMIA Open. 2019 Apr 26;2(2):261–71. doi: 10.1093/jamiaopen/ooz009 (PMC6951876; doi:10.1093/jamiaopen/ooz009)
Supplement: Supplement_Material_ooz009 [file supplement_material_ooz009.zip › APPENDIX 4redo.docx]

### APPENDIX 4 – PERFORMANCE OF DIFFERENT DEEP LEARNING BASED MODELS

Table 1 illustrates the performance of different deep learning models, trained on the training portion of the COPD corpus, tuned using the development set (see Tables 2-7 for details of the hyperparameters used for each model and Table 8 for the parameters used for initialising the Bayesian optimisation) and evaluated on the test set. In each case, the models are layered, and are evaluated on all entities (both nested and non-nested) in the test set of the corpus. Bi-directional versions of three deep learning models are evaluated, i.e., Recurrent Neural Network (BiRNN), Gated Recurrent Unit (BiGRU) and Long Short-Term Memory (BiLSTM). In each case, two different versions of the models were evaluated (i.e., alone and in combination with Conditional Random Fields (CRF)).

In all cases, the addition of CRF helped to boost performance, and the layered BiLSTM-CRF was the highest performing model.

**Table 1*.*** Performance of different layered deep learning based models applied to the test set of the COPD corpus.

| Models | P | R | F-score |
| --- | --- | --- | --- |
| Layered BiLSTM - CRF | 77.02 | 75.45 | 76.23 |
| Layered BiRNN - CRF | 75.72 | 67.52 | 71.38 |
| Layered BiGRU - CRF | 76.99 | 72.71 | 74.79 |
| Layered BiLSTM | 73.69 | 66.37 | 69.84 |
| Layered BiRNN | 64.58 | 53.62 | 58.59 |
| Layered BiGRU | 72.77 | 66.59 | 69.55 |

**Table 2.** Hyperparameters used in the layered BiLSTM-CRF model.

| **Parameters** | **Best value** | **Parameters** | **Best value** |
| --- | --- | --- | --- |
| Batch size | 121 | Dropout rate | 0.430745 |
| Learning rate | 0.008696 | Gradient clipping | 29 |
| Regularisation | 0.000293 |  |  |

**Table 3.** Hyperparameters used in the layered BiRNN-CRF model.

| **Parameters** | **Best value** | **Parameters** | **Best value** |
| --- | --- | --- | --- |
| Batch size | 92 | Dropout rate | 0.095110 |
| Learning rate | 0.004173 | Gradient clipping | 28 |
| Regularisation | 0.000592 |  |  |

**Table 4.** Hyperparameters used in the layered BiGRU-CRF model.

| **Parameters** | **Best value** | **Parameters** | **Best value** |
| --- | --- | --- | --- |
| Batch size | 65 | Dropout rate | 0.219963 |
| Learning rate | 0.004009 | Gradient clipping | 49 |
| Regularisation | 0.000861 |  |  |

**Table 5.** Hyperparameters used in the layered BiLSTM model.

| **Parameters** | **Best value** | **Parameters** | **Best value** |
| --- | --- | --- | --- |
| Batch size | 94 | Dropout rate | 0.330216 |
| Learning rate | 0.010222 | Gradient clipping | 16 |
| Regularisation | 0.000010 |  |  |

**Table 6.** Hyperparameters used in the layered BiRNN model.

| **Parameters** | **Best value** | **Parameters** | **Best value** |
| --- | --- | --- | --- |
| Batch size | 92 | Dropout rate | 0.095110 |
| Learning rate | 0.004173 | Gradient clipping | 28 |
| Regularisation | 0.000592 |  |  |

**Table 7.** Hyperparameters used in the layered BiGRU model.

| **Parameters** | **Best value** | **Parameters** | **Best value** |
| --- | --- | --- | --- |
| Batch size | 114 | Dropout rate | 0.359987 |
| Learning rate | 0.002182 | Gradient clipping | 29 |
| Regularisation | 0.000075 |  |  |

**Table 8.** Parameters used in initialising Bayesian optimisation.

| **Parameters** | **Initialisation value** | **Parameters** | **Initialisation value** |
| --- | --- | --- | --- |
| minimizer | gp_minimizer | noise | -1 |
| nCalls | 10 | acqOpt | lbfgs |
| randomstate | -1 | nRestartsOpt | 100 |
| acqFunc | gp_hedge | nPoints | 50000 |
| nRandomStarts | 10 | xi | 0.1 |
