# Supplementary material for: Annotating and detecting phenotypic information for chronic obstructive pulmonary disease
Source: JAMIA Open. 2019 Apr 26;2(2):261–71. doi: 10.1093/jamiaopen/ooz009 (PMC6951876; doi:10.1093/jamiaopen/ooz009)
Supplement: Supplement_Material_ooz009 [file supplement_material_ooz009.zip › APPENDIX 5.docx]

### APPENDIX 5 – DETAILED PERFORMANCE OF LAYERED MODEL

Table 1 illustrates the performance of the layered model for each semantic type annotated in the corpus. In Table 1, the performance of *ConstituentConcept* phenotype mentions is 100%. This is because there are no entities with this semantic type in the test data set and our model also did not predict any entities with this semantic type. In contrast, we obtained 0% performance for *MicrobiologicalTest* and *PhysiologicalTest* semantic types due to their sparsity in the testing data set.

**Table 1:** Performance of layered model on each semantic type.

| **Type** | **P (%)** | **R (%)** | **F (%)** | **# Entities** |
| --- | --- | --- | --- | --- |
| Problem | 70.54 | 69.00 | 69.76 | 229 |
| Condition | 86.75 | 86.57 | 86.66 | 484 |
| RiskFactor | 70.77 | 56.79 | 63.01 | 81 |
| SignOrSymptom | 60.38 | 53.63 | 56.81 | 179 |
| IndividualBehaviour | 63.64 | 77.78 | 70.00 | 9 |
| TestResult | 73.33 | 28.21 | 40.74 | 78 |
| Treatment | 81.66 | 81.34 | 81.50 | 509 |
| Test | 67.82 | 68.01 | 67.91 | 347 |
| RadiologicalTest | 50.00 | 25.00 | 33.33 | 4 |
| MicrobiologicalTest | 0 | 0 | 0 | 1 |
| PhysiologicalTest | 0 | 0 | 0 | 4 |
| ConstituentConcept | 100 | 100 | 100 | 0 |
| AnatomicalConcept | 74.09 | 84.33 | 78.88 | 217 |
| Drug | 85.15 | 87.36 | 86.24 | 348 |
| Protein | 63.89 | 63.01 | 63.45 | 73 |
| Quality | 76.87 | 76.87 | 76.87 | 134 |
